# Supplementary material for: Unstable Heteroresistance as a Stepping Stone to Stable Resistance via Compensatory Mutations
Source: bioRxiv. 2026 Oct 9:2026.10.08.756632. Preprint. [Version 1] doi: 10.64898/2026.10.08.756632 (PMC13692197; doi:10.64898/2026.10.08.756632)
Supplement: Supplementary file 1 [file NIHPP2026.10.08.756632v1-supplement-1.pdf]

bioRxiv preprint doi: https://doi.org/10.64898/2026.10.08.756632; this version posted October 9, 2026. The copyright holder for this preprint (which was not certified by peer review) is the author/funder, who has granted bioRxiv a license to display the preprint in perpetuity. It is made available under a CC-BY 4.0 International license.

Supplementary Materials for

Heteroresistance as a Stepping Stone to Resistance via Compensatory Mutations

Brandon A. Berryhill, Dan I. Andersson

bioRxiv preprint doi: https://doi.org/10.64898/2026.10.08.756632; this version posted October 9, 2026. The copyright holder for this preprint (which was not certified by peer review) is the author/funder, who has granted bioRxiv a license to display the preprint in perpetuity. It is made available under a CC-BY 4.0 International license.

Supplementary Text and Equations

Pharmacodynamics of Antibiotic Treatment

We assume there is a single limiting resource (*r*, µg/mL) which in the absence of antibiotics would be the sole limiting factor to the growth and ultimate density of the bacteria. The pharmacodynamics of the antibiotic and treated bacterial populations is given by a Hill function where *Π(A,r)* is the net growth/death rate of the bacteria [37]. Supplementary Equation 1 is written generally and applies to each of the twenty bacterial populations independently. To simulate the effect that the decreasing resource concentration has on the physiological state of the bacteria and their interactions, we include a term *ψ(r)* given in Supplementary Equation 2 [38].

$$\Pi(A,r) = \left(v - \frac{(v - v_{min}) \cdot \left(\frac{A}{MIC}\right)^{\kappa}}{\left(\frac{A}{MIC}\right)^{\kappa} - \frac{v_{min}}{v}} \right) \cdot \psi(r)$$

S. Eq 1

$$\psi(r) = \frac{r}{r + K}$$

S. Eq 2

Stochastic Transitions Between States

We simulate the transitions between states stochastically using a Monte Carlo process (18139350). A random number *x* (0 ≤ *x* ≤ 1) is generated from a rectangular distribution [39]. If *x* is less than the product of the number of cells in the generating state (e.g., R3, CFU/mL), the volume of the vessel (Vol, mL), the transition rate (µ for the most frequent

bioRxiv preprint doi: https://doi.org/10.64898/2026.10.08.756632; this version posted October 9, 2026. The copyright holder for this preprint (which was not certified by peer review) is the author/funder, who has granted bioRxiv a license to display the preprint in perpetuity. It is made available under a CC-BY 4.0 International license.

recombination events, γ for less frequent recombination events, or ω for compensatory mutations, all per generation per cell), and the step size (dt) of the Euler method employed for solving the differential equations then a transition occurs [40]. For example, if $x < R3 \cdot Vol \cdot \mu \cdot dt$, then MR3R2 cells are added to the R2 population and removed from the R3 population, where MR3R2 is 1/(dt·Vol). The same procedure is used for transitions between C states (e.g., MR3CR2C) and for compensation transitions between an R state and its C counterpart (e.g., MR3R3C and MR3CR3).

Heteroresistance Model

This model is adapted from the model presented in [10]. Briefly, the bacteria transition between twenty different states: R0 through R9, the densities (in cells per mL) of bacteria carrying 0 through 9 copies of β-lactamase gene, and R0C through R9C, the densities of their compensated counterparts which have additionally acquired a compensatory mutation that restores 50% of the fitness cost incurred by carrying the resistance gene. Transitions between R states occur stochastically as described above; transitions between C states occur via the same mechanism mirrored across the compensated space. The ability to transition between R states (or between C states) is limited by the copy number of the antibiotic-resistance gene present on each chromatid. The rate of transition is proportional to the number of ways recombination events can generate daughter cells with that copy number. Each state R*i* can additionally acquire a compensatory mutation at rate ω, transitioning to its compensated counterpart R*i*C, and revert at the same rate. A diagram of the model is presented in Supplementary Figure 2. The mechanism and rates of transition between R states (mirrored identically for the C

bioRxiv preprint doi: https://doi.org/10.64898/2026.10.08.756632; this version posted October 9, 2026. The copyright holder for this preprint (which was not certified by peer review) is the author/funder, who has granted bioRxiv a license to display the preprint in perpetuity. It is made available under a CC-BY 4.0 International license.

space) are reprinted from [10] in Supplementary Figure 1. This model assumes serial transfer conditions whereby all bacterial states and the antibiotic are diluted 1:100 every 24 hours, the β-lactamase is reset to zero, the limiting resource is reset to its maximum, and a fresh dose of antibiotic at concentration AMAX is delivered. Given these assumptions, the pharmacodynamics above, the definitions of the states (Supplementary Table 1), and the parameters and their values (Supplementary Table 2), the rates of change in the densities of the different populations are given by Supplementary Equation 3 through Supplementary Equation 25. β-lactamase production in Supplementary Equation 5 is scaled by the conversion efficiency *e*, the same physiological factor that sets resource consumption in Supplementary Equation 3.

$$\frac{dr}{dt} = -e \cdot \psi(r) \cdot (vR0 \cdot R0 + vR1 \cdot R1 + vR2 \cdot R2 + vR3 \cdot R3 + vR4 \cdot R4 + vR5 \cdot R5 + vR6 \cdot R6 + vR7 \cdot R7 + vR8 \cdot R8 + vR9 \cdot R9 + vR0C \cdot R0C + vR1C \cdot R1C + vR2C \cdot R2C + vR3C \cdot R3C + vR4C \cdot R4C + vR5C \cdot R5C + vR6C \cdot R6C + vR7C \cdot R7C + vR8C \cdot R8C + vR9C \cdot R9C$$

S. Eq 3

$$\frac{dA}{dt} = -d \cdot A - KB \cdot B \cdot A$$

S. Eq 4

$$\frac{dB}{dt} = e \cdot \psi(r) \cdot (pR0 \cdot R0 + pR1 \cdot R1 + pR2 \cdot R2 + pR3 \cdot R3 + pR4 \cdot R4 + pR5 \cdot R5 + pR6 \cdot R6 + pR7 \cdot R7 + pR8 \cdot R8 + pR9 \cdot R9 + pR0C \cdot R0C + pR1C \cdot R1C + pR2C \cdot R2C + pR3C \cdot R3C + pR4C \cdot R4C + pR5C \cdot R5C + pR6C \cdot R6C + pR7C \cdot R7C + pR8C \cdot R8C + pR9C \cdot R9C)$$

bioRxiv preprint doi: https://doi.org/10.64898/2026.10.08.756632; this version posted October 9, 2026. The copyright holder for this preprint (which was not certified by peer review) is the author/funder, who has granted bioRxiv a license to display the preprint in perpetuity. It is made available under a CC-BY 4.0 International license.

S. Eq 5

$$\frac{dR0}{dt} = R0 \cdot \Pi_{R0}(A,r) + (MR10 + MR20 + MR30 + MR40 + MR50 + MR60 + MR70 + MR80 + MR90 + MR0CR0 - MR0R0C$$

S. Eq 6

$$\frac{dR1}{dt} = R1 \cdot \Pi_{R1}(A,r) + (MR21 + MR31 + MR41 + MR51 + MR61 + MR71 + MR81 + MR91 - MR10 + MR12 + MR1CR1 - MR1R1C$$

S. Eq 7

$$\frac{dR2}{dt} = R2 \cdot \Pi_{R2}(A,r) + (MR12 + MR32 + MR42 + MR52 + MR62 + MR72 + MR82 + MR92 - MR20 + MR21 + MR23 + MR24 + MR2CR2 - MR2R2C$$

S. Eq 8

$$\frac{dR3}{dt} = R3 \cdot \Pi_{R3}(A,r) + (MR23 + MR43 + MR53 + MR63 + MR73 + MR83 + MR93 - MR30 + MR31 + MR32 + MR34 + MR35 + MR36 + MR3CR3 - MR3R3C$$

S. Eq 9

$$\frac{dR4}{dt} = R4 \cdot \Pi_{R4}(A,r) + (MR24 + MR34 + MR54 + MR64 + MR74 + MR84 + MR94 - MR40 + MR41 + MR42 + MR43 + MR45 + MR46 + MR47 + MR48 + MR4CR4 - MR4R4C$$

S. Eq 10

bioRxiv preprint doi: https://doi.org/10.64898/2026.10.08.756632; this version posted October 9, 2026. The copyright holder for this preprint (which was not certified by peer review) is the author/funder, who has granted bioRxiv a license to display the preprint in perpetuity. It is made available under a CC-BY 4.0 International license.

$$\frac{dR5}{dt} = R5 \cdot \Pi_{R5}(A,r) + (MR35 + MR45 + MR65 + MR75 + MR85 + MR95) - (MR50 + MR51 + MR52 + MR53 + MR54 + MR56 + MR57 + MR58 + MR59) + MR5CR5 - MR5R5C$$

S. Eq 11

$$\frac{dR6}{dt} = R6 \cdot \Pi_{R6}(A,r) + (MR36 + MR46 + MR56 + MR76 + MR86 + MR96) - (MR60 + MR61 + MR62 + MR63 + MR64 + MR65 + MR67 + MR68 + MR69) + MR6CR6 - MR6R6C$$

S. Eq 12

$$\frac{dR7}{dt} = R7 \cdot \Pi_{R7}(A,r) + (MR47 + MR57 + MR67 + MR87 + MR97) - (MR70 + MR71 + MR72 + MR73 + MR74 + MR75 + MR76 + MR78 + MR79 + MR7CR7 - MR7R7C$$

S. Eq 13

$$\frac{dR8}{dt} = R8 \cdot \Pi_{R8}(A,r) + (MR48 + MR58 + MR68 + MR78 + MR98) - (MR80 + MR81 + MR82 + MR83 + MR84 + MR85 + MR86 + MR87 + MR89 + MR8CR8 - MR8R8C$$

S. Eq 14

$$\frac{dR9}{dt} = R9 \cdot \Pi_{R9}(A,r) + (MR59 + MR69 + MR79 + MR89) - (MR90 + MR91 + MR92 + MR93 + MR94 + MR95 + MR96 + MR97 + MR98 + MR9CR9 - MR9R9C$$

S. Eq 15

bioRxiv preprint doi: https://doi.org/10.64898/2026.10.08.756632; this version posted October 9, 2026. The copyright holder for this preprint (which was not certified by peer review) is the author/funder, who has granted bioRxiv a license to display the preprint in perpetuity. It is made available under a CC-BY 4.0 International license.

$$\frac{dR0C}{dt} = R0C \cdot \Pi_{R0C}(A,r) + (MR1CR0C + MR2CR0C + MR3CR0C + MR4CR0C + MR5CR0C + MR6CR0C + MR7CR0C + MR8CR0C + MR9CR0C + MR0R0C - MR0CR0$$

S. Eq 16

$$\frac{dR1C}{dt} = R1C \cdot \Pi_{R1C}(A,r) + (MR2CR1C + MR3CR1C + MR4CR1C + MR5CR1C + MR6CR1C + MR7CR1C + MR8CR1C + MR9CR1C - MR1CR0C + MR1CR2C + MR1R1C - MR1CR1$$

S. Eq 17

$$\frac{dR2C}{dt} = R2C \cdot \Pi_{R2C}(A,r) + (MR1CR2C + MR3CR2C + MR4CR2C + MR5CR2C + MR6CR2C + MR7CR2C + MR8CR2C + MR9CR2C - MR2CR0C + MR2CR1C + MR2CR3C + MR2CR4C + MR2R2C - MR2CR2$$

S. Eq 18

$$\frac{dR3C}{dt} = R3C \cdot \Pi_{R3C}(A,r) + (MR2CR3C + MR4CR3C + MR5CR3C + MR6CR3C + MR7CR3C + MR8CR3C + MR9CR3C - MR3CR0C + MR3CR1C + MR3CR2C + MR3CR4C + MR3CR5C + MR3CR6C + MR3R3C - MR3CR3$$

S. Eq 19

$$\frac{dR4C}{dt} = R4C \cdot \Pi_{R4C}(A,r) + (MR2CR4C + MR3CR4C + MR5CR4C + MR6CR4C + MR7CR4C + MR8CR4C + MR9CR4C) - (MR4CR0C + MR4CR1C + MR4CR2C + MR4CR3C + MR4CR5C + MR4CR6C + MR4CR7C + MR4CR8C) + MR4R4C - MR4CR4$$

bioRxiv preprint doi: https://doi.org/10.64898/2026.10.08.756632; this version posted October 9, 2026. The copyright holder for this preprint (which was not certified by peer review) is the author/funder, who has granted bioRxiv a license to display the preprint in perpetuity. It is made available under a CC-BY 4.0 International license.

S. Eq 20

$$\frac{dR5C}{dt} = R5C \cdot \Pi_{R5C}(A,r) + (MR3CR5C + MR4CR5C + MR6CR5C + MR7CR5C + MR8CR5C + MR9CR5C - MR5CR0C + MR5CR1C + MR5CR2C + MR5CR3C + MR5CR4C + MR5CR6C + MR5CR7C + MR5CR8C + MR5CR9C + MR5R5C - MR5CR5$$

S. Eq 21

$$\frac{dR6C}{dt} = R6C \cdot \Pi_{R6C}(A,r) + (MR3CR6C + MR4CR6C + MR5CR6C + MR7CR6C + MR8CR6C + MR9CR6C - MR6CR0C + MR6CR1C + MR6CR2C + MR6CR3C + MR6CR4C + MR6CR5C + MR6CR7C + MR6CR8C + MR6CR9C + MR6R6C - MR6CR6$$

S. Eq 22

$$\frac{dR7C}{dt} = R7C \cdot \Pi_{R7C}(A,r) + (MR4CR7C + MR5CR7C + MR6CR7C + MR8CR7C + MR9CR7C - MR7CR0C + MR7CR1C + MR7CR2C + MR7CR3C + MR7CR4C + MR7CR5C + MR7CR6C + MR7CR8C + MR7CR9C + MR7R7C - MR7CR7$$

S. Eq 23

$$\frac{dR8C}{dt} = R8C \cdot \Pi_{R8C}(A,r) + (MR4CR8C + MR5CR8C + MR6CR8C + MR7CR8C + MR9CR8C - MR8CR0C + MR8CR1C + MR8CR2C + MR8CR3C + MR8CR4C + MR8CR5C + MR8CR6C + MR8CR7C + MR8CR9C + MR8R8C - MR8CR8$$

S. Eq 24

bioRxiv preprint doi: https://doi.org/10.64898/2026.10.08.756632; this version posted October 9, 2026. The copyright holder for this preprint (which was not certified by peer review) is the author/funder, who has granted bioRxiv a license to display the preprint in perpetuity. It is made available under a CC-BY 4.0 International license.

$$\frac{dR9C}{dt} = R9C \cdot \Pi_{R9C}(A,r) + (MR5CR9C + MR6CR9C + MR7CR9C + MR8CR9C) - (MR9CR0C + MR9CR1C + MR9CR2C + MR9CR3C + MR9CR4C + MR9CR5C + MR9CR6C + MR9CR7C + MR9CR8C + MR9R9C - MR9CR9$$

S. Eq 25

bioRxiv preprint doi: https://doi.org/10.64898/2026.10.08.756632; this version posted October 9, 2026. The copyright holder for this preprint (which was not certified by peer review) is the author/funder, who has granted bioRxiv a license to display the preprint in perpetuity. It is made available under a CC-BY 4.0 International license.

Supplementary Figures

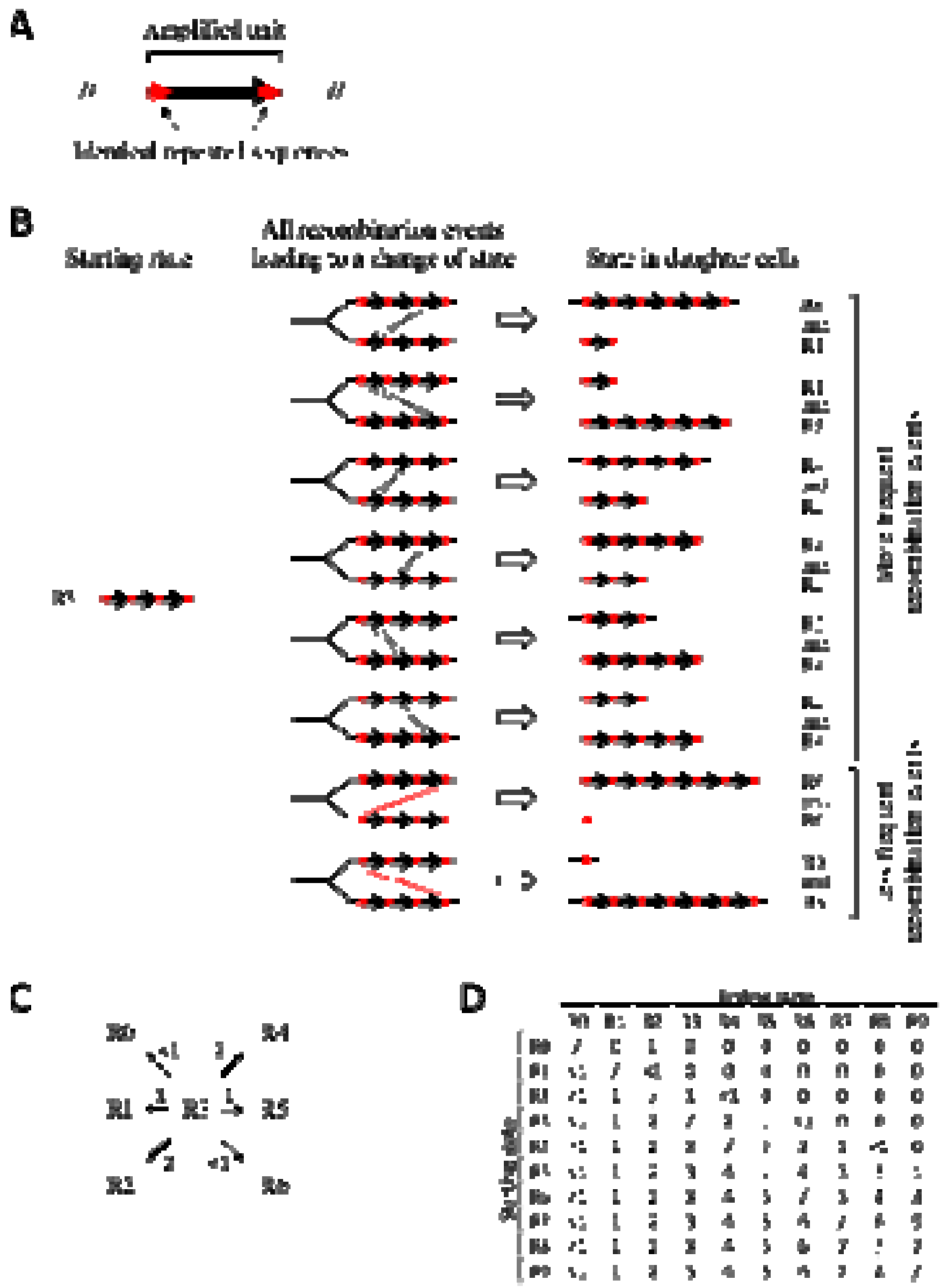

Supplementary Figure 1. Transition and rates of transitions and mechanism within R states and RC states. Reproduced with permission from [10]. A) General structure of the amplified unit in our model. The amplified unit (often several kilobases in length) contains identical repeated sequences at each end (e.g., IS sequences <1 kb in length). **B)** Example, for cells starting with a triplication of the amplified unit (state R3), of all recombination events between amplified units present on sister chromatids and leading to a transition to other states in daughter cells. The more frequent recombinations occur between the amplified units (in black) while less frequent recombinations involve shorter identical repeats that are present at each end of the array of amplified units (in red). **C)** Summary of results presented in panel B schematizing the relative rates of transition between R states (arbitrary unit) for a cell in a starting state R3. **D)** All relative rates of transition between R states (arbitrary unit) for our model, for starting and ending R states constrained between R0 and R9.

bioRxiv preprint doi: https://doi.org/10.64898/2026.10.08.756632; this version posted October 9, 2026. The copyright holder for this preprint (which was not certified by peer review) is the author/funder, who has granted bioRxiv a license to display the preprint in perpetuity. It is made available under a CC-BY 4.0 International license.

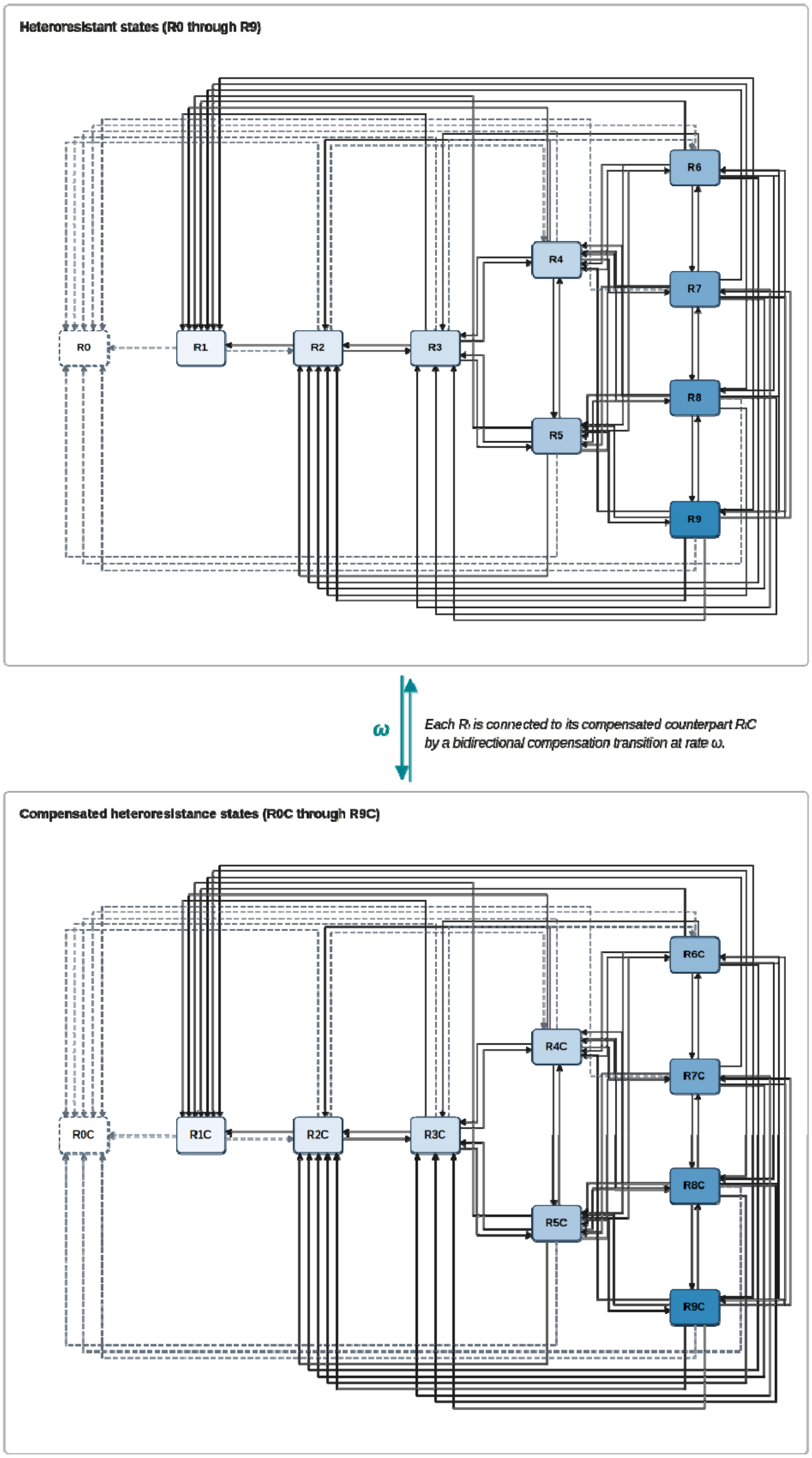

bioRxiv preprint doi: https://doi.org/10.64898/2026.10.08.756632; this version posted October 9, 2026. The copyright holder for this preprint (which was not certified by peer review) is the author/funder, who has granted bioRxiv a license to display the preprint in perpetuity. It is made available under a CC-BY 4.0 International license.

Supplementary Figure 2. Diagram of the Model. The model comprises twenty bacterial states. R states (R0 to R9) correspond to bacteria with increasing copy number of a tandem amplified resistance gene, ranging from 0 to 9 copies; the compensated states (R0C to R9C) carry the same copy number but have additionally acquired a compensatory mutation. Solid arrows denote the more frequent recombination events (γ); dashed arrows denote the less frequent events (μ). Recombination occurs identically within the R space and within the C space. Teal arrows denote the compensation transition (ω) where each R state acquires a compensatory mutation, transitioning to its compensated counterpart. The model includes all possible transitions between states for starting states R0 to R9. The relative rates of all transitions are given in Supplementary Figure 1 and Supplementary Table 2.

bioRxiv preprint doi: https://doi.org/10.64898/2026.10.08.756632; this version posted October 9, 2026. The copyright holder for this preprint (which was not certified by peer review) is the author/funder, who has granted bioRxiv a license to display the preprint in perpetuity. It is made available under a CC-BY 4.0 International license.

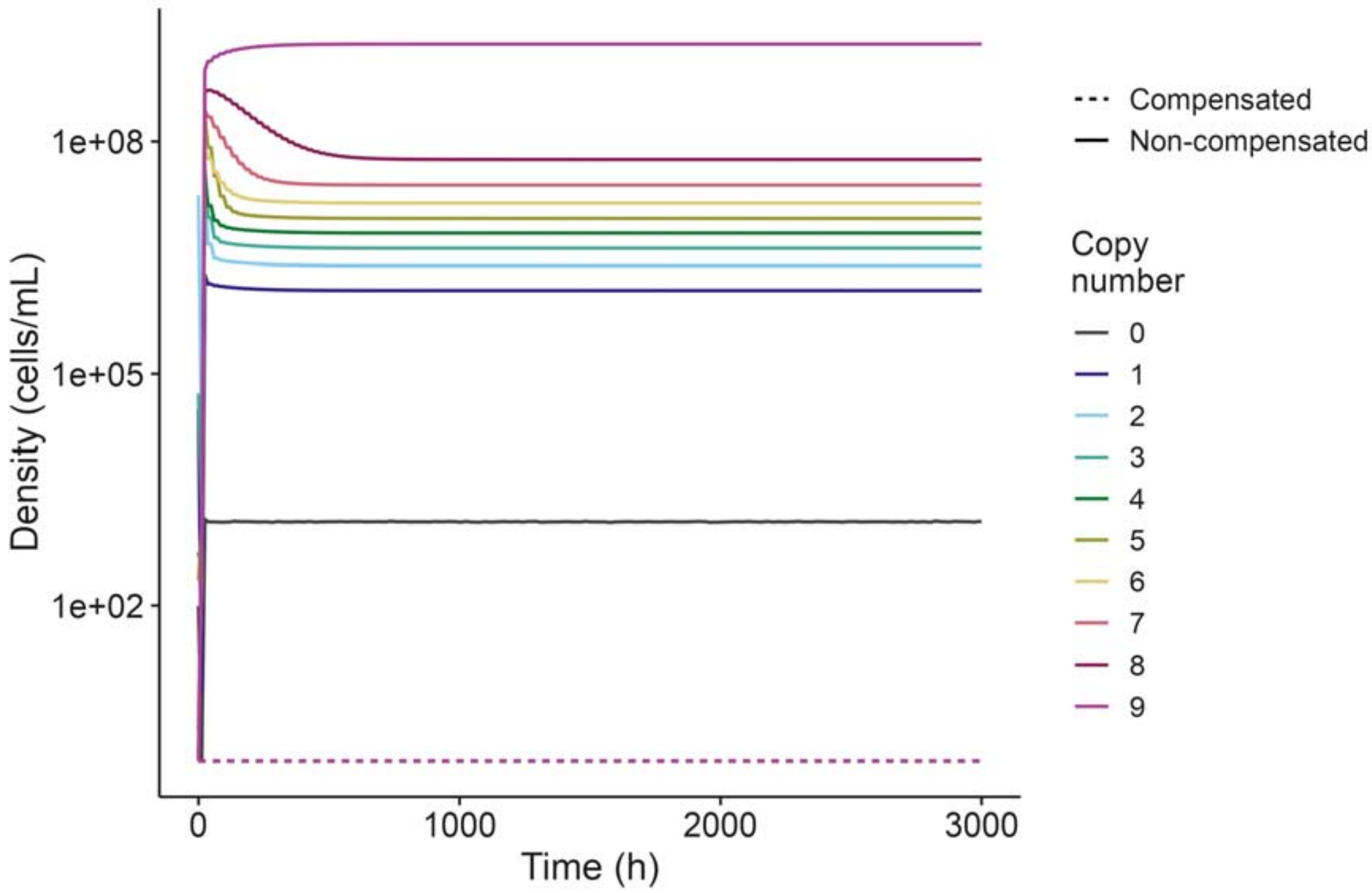

Supplementary Figure 3. Dynamics of the model in the absence of compensatory mutations. The model was run under the conditions of Figure 1 with the compensation rate set to zero (ω = 0). The densities of the non-compensated states (R0 through R9) and the compensated states (R0C through R9C) are shown across the full 3,000 h simulation. The compensated states remain at zero, while the non-compensated states evolve by recombination alone and recapitulate the base model [10]. Lines are coloured by copy number, with non-compensated states drawn solid and compensated states dashed.

bioRxiv preprint doi: https://doi.org/10.64898/2026.10.08.756632; this version posted October 9, 2026. The copyright holder for this preprint (which was not certified by peer review) is the author/funder, who has granted bioRxiv a license to display the preprint in perpetuity. It is made available under a CC-BY 4.0 International license.

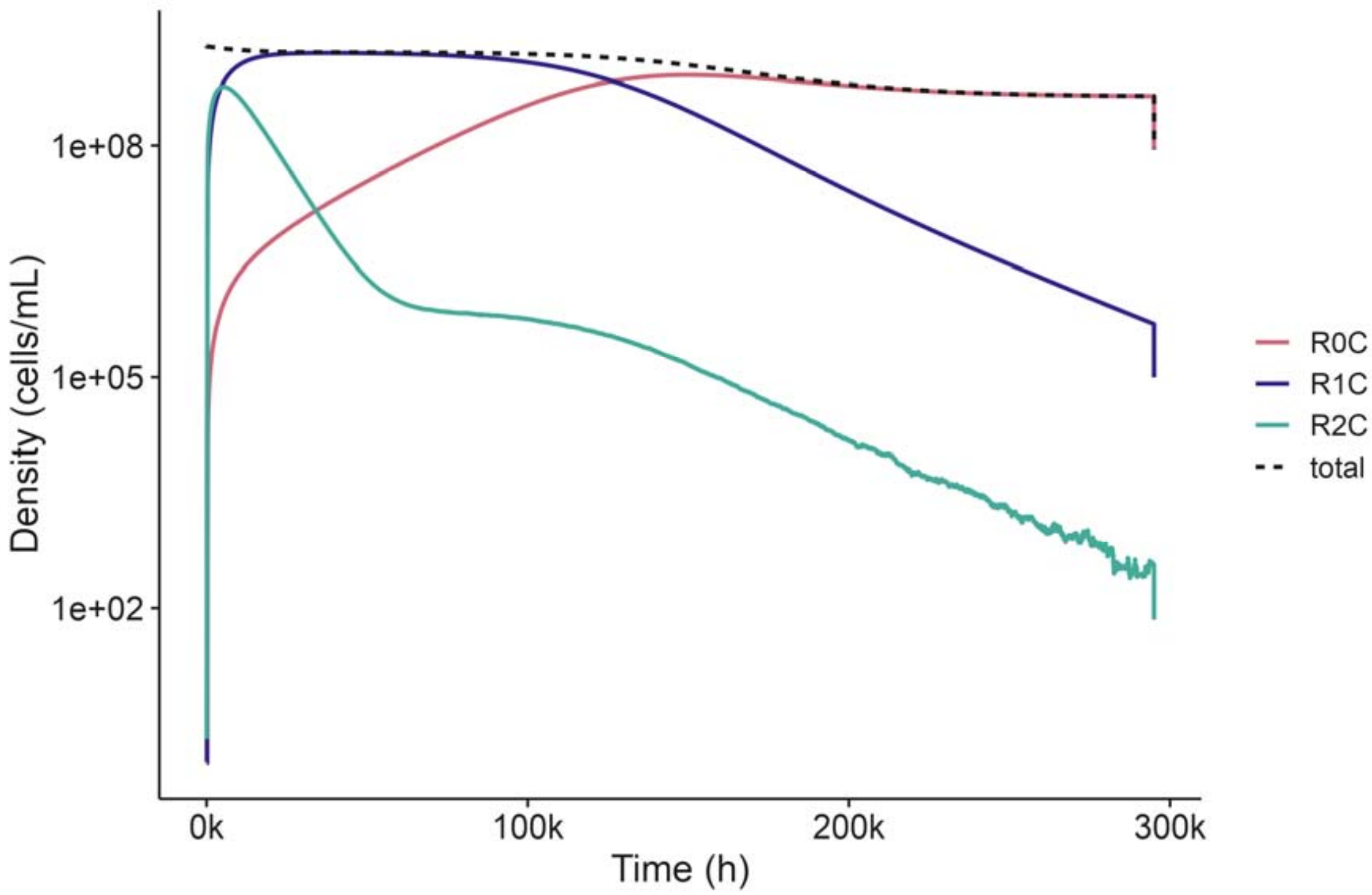

Supplementary Figure 4. Far-horizon predictions over 300,000 hours. The deterministic model was run to 300,000 h to follow the final approach to the resistant endpoint. The three lowest compensated states, R0C, R1C, and R2C, and the total population (dashed black) are shown. The two-copy state R2C declines first, the single-copy state R1C dominates the intermediate horizon, and the zero-copy state R0C, the resistant endpoint that carries no amplification, overtakes R1C near 126,000 h—over 14 years, well beyond any clinically-relevant timescale.

bioRxiv preprint doi: https://doi.org/10.64898/2026.10.08.756632; this version posted October 9, 2026. The copyright holder for this preprint (which was not certified by peer review) is the author/funder, who has granted bioRxiv a license to display the preprint in perpetuity. It is made available under a CC-BY 4.0 International license.

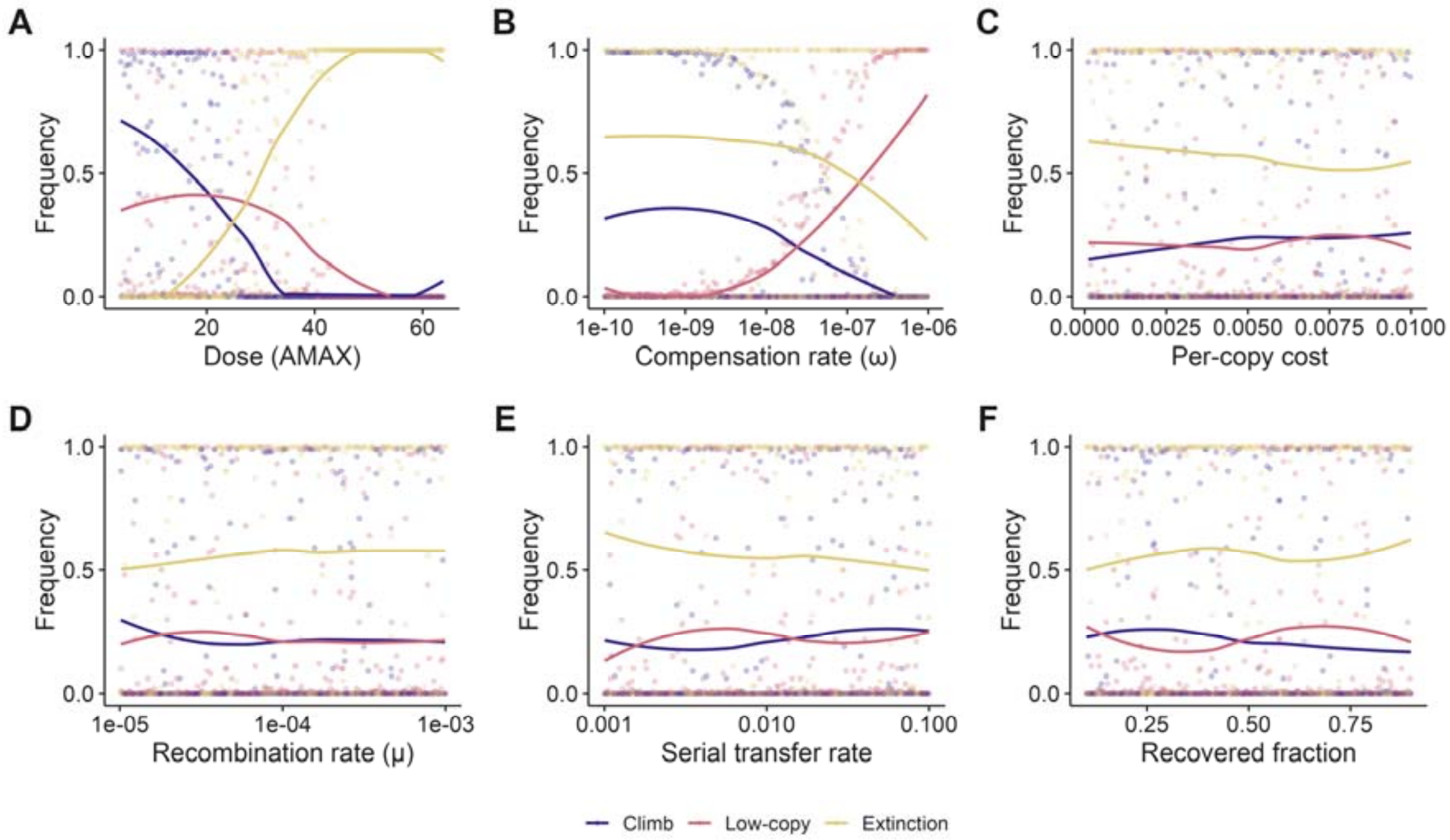

Supplementary Figure 5. The raw parameter sweep. Each panel shows one of the six swept parameters on the x axis against the frequency of the three outcomes: climb, low-copy compensated survival, and extinction. Points are individual parameter sets and lines are smoothed trends. Dose drives extinction; the compensation rate governs the two survival routes; and the per-copy cost, recombination rate, serial transfer rate, and recovered fraction have little effect.

bioRxiv preprint doi: https://doi.org/10.64898/2026.10.08.756632; this version posted October 9, 2026. The copyright holder for this preprint (which was not certified by peer review) is the author/funder, who has granted bioRxiv a license to display the preprint in perpetuity. It is made available under a CC-BY 4.0 International license.

Supplementary Tables

Supplementary Table 1. States in the Model

State	Dimensions	Definition
r	µg per mL	Limiting Resource
R0	Cells per mL	Antibiotic-susceptible Bacteria (Zero Copies of Resistance Gene)
R1, R2, R3, R4, R5, R6, R7, R8, R9	Cells per mL	Non-compensated Antibiotic-resistant Bacteria with Increasing Copies of Resistance Gene
R0C	Cells per mL	Compensated Antibiotic-susceptible Bacteria (Zero Copies of Resistance Gene)
R1C, R2C, R3C, R4C, R5C, R6C, R7C, R8C, R9C	Cells per mL	Compensated Antibiotic-resistant Bacteria with Increasing Copies of Resistance Gene
A	µg per mL	Antibiotic

bioRxiv preprint doi: https://doi.org/10.64898/2026.10.08.756632; this version posted October 9, 2026. The copyright holder for this preprint (which was not certified by peer review) is the author/funder, who has granted bioRxiv a license to display the preprint in perpetuity. It is made available under a CC-BY 4.0 International license.

B	Units per mL	β-lactamase

bioRxiv preprint doi: https://doi.org/10.64898/2026.10.08.756632; this version posted October 9, 2026. The copyright holder for this preprint (which was not certified by peer review) is the author/funder, who has granted bioRxiv a license to display the preprint in perpetuity. It is made available under a CC-BY 4.0 International license.

741 **Supplementary Table 2. Parameters in the Model**

Parameter	Dimensions	Definition	Values	Source
vR0, vR1, vR2, vR3, vR4, vR5, vR6, vR7, vR8, vR9	Per Cell Per Hour	Maximum Growth Rates	2, 1.9987, 1.9974, 1.9961, 1.9948, 1.9935, 1.9922, 1.9909, 1.9896, 1.9883	[10]
vR0C, vR1C, vR2C, vR3C, vR4C, vR5C, vR6C, vR7C, vR8C, vR9C	Per Cell Per Hour	Maximum Growth Rates of Compensated States	2, 1.99935, 1.9987, 1.99805, 1.9974, 1.99675, 1.9961, 1.99545, 1.9948, 1.99415	This Study

bioRxiv preprint doi: https://doi.org/10.64898/2026.10.08.756632; this version posted October 9, 2026. The copyright holder for this preprint (which was not certified by peer review) is the author/funder, who has granted bioRxiv a license to display the preprint in perpetuity. It is made available under a CC-BY 4.0 International license.

μRx, γRx (frequent and rarer transitions)	Per Cell Per Hour	Recombination Transition Rates	$4 \bullet 10^{-4}$ to $4 \bullet 10^{-7}$	[10]
ω	Per Cell Per Hour	Compensation Rate	$1 \bullet 10^{-7}$	This Study
vMIN	Per Cell Per Hour	Minimum Growth/Death Rate	-2	[41]
κ	Dimensionless	Hill Coefficient	1	[37]
MICR0, MICR1, MICR2, MICR3, MICR4, MICR5, MICR6, MICR7, MICR8, MICR9	μg per mL	MIC of Each State	0.375, 1.875, 3.375, 4.875, 6.375, 7.875, 9.375, 10.875, 12.375, 13.875	[10]
MICR0C, MICR1C, MICR2C, MICR3C,	μg per mL	MIC of Each Compensated State, Scenario 1	25, 25, 25, 25, 25,	This Study

bioRxiv preprint doi: https://doi.org/10.64898/2026.10.08.756632; this version posted October 9, 2026. The copyright holder for this preprint (which was not certified by peer review) is the author/funder, who has granted bioRxiv a license to display the preprint in perpetuity. It is made available under a CC-BY 4.0 International license.

MICR4C, MICR5C, MICR6C, MICR7C, MICR8C, MICR9C			25, 25, 25, 25, 25	
MICR0C, MICR1C, MICR2C, MICR3C, MICR4C, MICR5C, MICR6C, MICR7C, MICR8C, MICR9C	µg per mL	MIC of Each Compensated State, Scenario 2	1.125, 5.625, 10.125, 14.625, 19.125, 23.625, 28.125, 32.625, 37.125, 41.625	This Study
pR0, pR1, pR2, pR3, pR4, pR5,	Units per Hour	β-lactamase Production Rate	0.000, 0.001, 0.002, 0.003, 0.004, 0.005, 0.006, 0.007, 0.008, 0.009	[10]

bioRxiv preprint doi: https://doi.org/10.64898/2026.10.08.756632; this version posted October 9, 2026. The copyright holder for this preprint (which was not certified by peer review) is the author/funder, who has granted bioRxiv a license to display the preprint in perpetuity. It is made available under a CC-BY 4.0 International license.

pR6, pR7, pR8, pR9				
pR0C, pR1C, pR2C, pR3C, pR4C, pR5C, pR6C, pR7C, pR8C, pR9C	Units per Hour	β-lactamase Production Rate of Compensated States	0.000, 0.001, 0.002, 0.003, 0.004, 0.005, 0.006, 0.007, 0.008, 0.009	This Study
KB	Dimensionless	β-lactamase Association Constant	1	[10]
k	Dimensionless	Monod Constant	1	[42]
e	µg	Conversion Efficiency	$5 \bullet 10^{-7}$	[42]
C	µg per mL	Maximum Resource	1000	Assumed
d	µg per Hour	Antibiotic Decay	0	Assumed

bioRxiv preprint doi: https://doi.org/10.64898/2026.10.08.756632; this version posted October 9, 2026. The copyright holder for this preprint (which was not certified by peer review) is the author/funder, who has granted bioRxiv a license to display the preprint in perpetuity. It is made available under a CC-BY 4.0 International license.

		Rate		
AMAX	µg per mL	Antibiotic Dose at Each Transfer	16	This Study
dil	Dimensionless	Serial Transfer Rate	0.01	This Study
